# Does measurement invariance hold for the official Mexican multidimensional poverty measure? A state-level analysis 2012

**DOI:** 10.1007/s11135-016-0327-0

**Published:** 2016-03-17

**Authors:** Hector Ernesto Najera

**Affiliations:** School for Policy Studies, Centre for the Study of Poverty and Social Justice, University of Bristol, Bristol, UK

**Keywords:** Measurement invariance, Poverty, Measurement

## Abstract

One of the main goals in poverty measurement is making comparisons of prevalence and severity across geographical units. This is attained by merely disaggregating the index in question. The underlying assumption is that comparisons across units are tenable, inasmuch as the same indicators are utilised for constructing the index. Nonetheless, in practice, this assumption is very rarely tested. From the statistical perspective, measurement invariance (MI) must hold for comparisons to be valid, and violations thereof indicate that a given poverty index measures different things across different countries, states, counties, etc. Consequently, differentials in severity and prevalence cannot be attributed exclusively to the underlying construct (i.e. poverty) but to factors not considered in the measure. This article tests whether MI holds for two indexes: the Mexican official multidimensional measure (MPM) and an adjusted multidimensional measure (MPM-A) that uses less severe thresholds. The analysis is conducted using a novel method called the ‘alignment method’. It uses these two measures and the method as an illustration of why it is vital to introduce MI tests into poverty measurement. The results suggest that partial strong MI holds for the official measure and MI is violated when the thresholds are adjusted. Partial strong MI guarantees making valid comparisons across the 32 states. Should the official measure requires to be updated with other thresholds, it would be necessary to adjust the threshold or drop the indicator for water deprivation.

## Introduction

In poverty studies, there is often interest in comparing deprivation prevalence and/or severity across countries, regions, states, smaller geographical areas and/or population groups. The aim of such exercises is to assess where or who has the higher/lower prevalence and/or severity rate. This task is often undertaken after producing a poverty index, and it consists in disaggregating the measure in question for different geographical units or groups. Such comparisons are vital from a policy perspective, as such rankings are used as a reference for targeting resources to specific regions or areas, in order to tackle poverty. Likewise, showing disadvantages across countries and regions is decisive for international and local policy design and implementation.

Poverty research has relied on disaggregation as means to produce poverty estimates for different population groups and countries (Alkire and Foster 2011b; Alkire and Santos 2010; Guio et al. 2012; Whelan et al. 2014). The underlying assumption is that poverty is measured equivalently across populations, i.e. the indicators utilized to construct a multidimensional index are invariant manifestations of poverty across the groups or countries of interest. Nonetheless, unlike in other fields in social sciences and psychometrics (Byrne et al. 1989; Meredith 1993; van de Schoot et al. 2012), the implications of lacking a comparable measure have not been thoroughly discussed.ournal instruction requires a city and country for affiliations; however, these are missing in affiliation Please verify if the provided city and country are correct and amend if necessary.

Multidimensional poverty measures rely on series of indicators to produce an index. They are combined in some way to compute a synthetic estimate of poverty. However, these indicators should be comparable in order to result in a valid and comparable measure across countries or groups. Otherwise comparisons of specific deprivations are unlikely to be tenable. This opens up questions regarding disaggregation and comparability in poverty research: Is it valid to compare two or more countries/groups using the proposed index? Are there any indicators that are particularly problematic to make comparisons? Is poverty in country *j* really higher than in country *k*? What are the effects upon comparability of using different collection modes, samples and countries with different standards of living? How, from a methodological perspective, is it possible to examine and correct this problem?

From statistical perspective having a comparable measure means that the indicators fulfil *Measurement Invariance* (MI), i.e. they measure poverty equivalently across the groups of interest (Meredith 1993). One of the reasons why this crucial assumption has remained untested is methodological. The psychometric literature now enable to examine MI using large samples, which are quite common in poverty research been very concerned with comparability issues, i.e. whether a test is useful to examine the level of skill, intelligence, depression, addiction, etc. across different populations (Meredith 1993; Byrne et al. 1989). The statistical theory for assessing MI can be traced back to the late 1980s, although the computational capabilities to take MI examinations further is fairly recent (Muthén 1989; Meredith 1993). Moreover, further refinements to MI theory offer the possibility of conducting more flexible and ambitious analyses, i.e. testing MI for a large set of sub-populations (Asparouhov and Muthén 2014).

The main goals of this article are to discuss why MI should be a concern in poverty research, describe the implications of violating MI in the context of poverty studies and to show a way to assess it using novel statistical methods. It uses as a motivating example, namely the official Mexican poverty measure (MPM). As with many other measures, the MPM uses disaggregation as a means of comparing and ranking Mexican states and municipalities according to the prevalence of poverty (CONEVAL 2011b). However, it remains unknown as to whether MI holds for this measure when using states as a grouping variable. Moreover, this measure, as many others, will demand to be updated and it is unclear whether using less severe cut-offs results in violating MI. This will be useful to explore the assumption that using acute or absolute poverty is the best approach to performing comparisons across groups, in this case the 32 Mexican states.

The article is organised as follows. The first section outlines the implications of ignoring and then violating measurement invariance in the context of poverty research, following which the second section presents from a statistical perspective the main features of MI. The third section describes the data utilised for the analysis, and the fourth section shows the results of the analysis. The fifth section discusses and concludes the article.

## Poverty measurement and measurement invariance

One of the main goals in poverty measurement is analysing how the prevalence or severity of poverty varies across different sub-populations and to understand why, i.e. what are the predictors of poverty(A few example sinclude Whelan et al. 2014; Battiston et al. 2013; Fusco et al. 2013; Alkire and Santos 2010; Anand et al. 2010; Dorling et al. 2007). Group membership is usually defined by using demographic characteristics such as gender, age, ethnicity or place (country, state, region, etc.). In other cases, there is also the question about whether a scale is comparable across time, i.e. whether the same index can be utilized to compare severity of poverty between 1990 and 2010, for example. When using the same measure to contrast prevalence or severity rates between groups, finding that the mean deprivation score in country *j* is higher than the mean in country *k* is often regarded as proof enough to conclude that the severity of deprivation is higher in *j* with respect *k*.

Disaggregation has been used since classic poverty studies as a means of contrasting poverty rates across groups and geographical units (Rowntree 1901; Townsend 1979). The prevalence rates for different groups are therefore an outcome of merely dividing the dataset according to groups of interest. The underlying assumption supporting the validity of disaggregation, in international comparative studies for example, seems to be that using the same indicators for different groups guarantees a certain degree of comparability (Alkire and Foster 2011b; Alkire and Santos 2010; Guio et al. 2012; Whelan et al. 2014). Some frameworks even consider disaggregation as a property rather than an assumption (Alkire and Foster 2011a). Nevertheless, disaggregation is not an inherent theoretical and statistical property of a measure; instead, it is just a consequence of dividing the matrices of indicators.

From a theoretical perspective, much of the discussion about comparability has revolved around the question about how to compare countries with different standards of living or preferences. According to Townsend (1979), poverty is relative across time and space, and the theory of relative deprivation suggests that for poverty to be compared across two countries, for example, it is necessary to take into consideration the standard of living of both nations and then to develop a measure that permits valid comparisons using the same metric (Gordon 2006).

One presumption in the literature is that comparability can be attained by using the society with the lowest standard of living as a reference, i.e. a country is poorer is relative to other. This approach has been adopted by the United Nations Development Programme (UNDP) and by the Economic Council for Latin America and the Caribbean (ECLAC) (UNDP 2014; CEPAL 2013). This is approach akin to the idea of invoking the notion of absolute or acute poverty, which became an internationally accepted concept after the World Summit for Social Development in 1995 (UN 1995; Townsend and Gordon 2000; Alkire and Santos 2010). This concept refers to the use of a minimum core of human needs and it can be related with the severest forms of material deprivation (Gordon 2006). Therefore, such a minimum core should be equivalent across societies, making poverty rates comparable. However, this just opens an empirical question. If, in theory, an absolute measure is capable of offering a better basis for cross-group comparisons, this must be backed up by empirical research.

Differences in standard of living and/or preferences, as in the socially perceived necessities approach, pose challenges to produce valid, reliable and comparable measures across countries and populations. But there are other problems with potential negative implications like error measurement (Vannieuwenhuyze et al. 2010; Vannieuwenhuyze 2015). Multidimensional poverty measurement relies on data collected using surveys, which utilize different sampling frameworks, data collection modes, translated/adapted questionnaires, and so forth. Although harmonized data attempts to mitigate the negative effects of these issues, in practice is rarely tested whether the deprivation indicators are comparable.

One way to frame, from a statistical perspective, the question about comparability is by adopting the concept of measurement invariance in poverty research. MI is a key assumption in modern psychometric theory (Meredith and Teresi 2006; Meredith 1993), and it means that a given scale is equivalent across groups. In other words, it means measuring the same thing (latent construct, in this case poverty) on the same basis across sub-populations. A common example of MI is a test that aims at comparing the educational achievement of students across countries. Such a test is invariant when the questions evaluate on an equal basis Spanish-speaking students and English-speaking students, i.e. the country, or more precisely their language, is not favoured by the test. Should such a test be found non-invariant, it is likely that an unknown factor will not be accounted for by the model; for example, the ability to speak Spanish when the test favours this population group.

How can this rationale be extended to poverty research? Measurement invariance states that using the same indicators might be necessary but not sufficient to make comparisons. There is a potential difference between using the *same* and using a *suitable* set of indicators. Suitability is a necessary condition for comparability, and the only way to achieve it is when *the same underlying model* holds for the sub-populations in question. To grasp what this means, it is important to consider modern psychometric theory (also known as the ‘latent variable’ approach) (Keith 2015; Brown 2006). Within this framework, indicators are manifestations of an underlying phenomenon, the consequences of which are observed (e.g. lacking clean water, is one of many manifestations of overall deprivation).

When the same model does not hold for different sub-populations, it means that the relationship between overall deprivation (underlying phenomenon) and the observed variables (unmet necessities for modern life) is not equivalent across groups. A non-equivalent model denotes a situation where (1) changes in overall deprivation (slope in a regression model) are unevenly associated with observed deprivation across groups and/or (2) the starting point (intercept in a regression model) of two groups regarding observed deprivations is different. An example of the first case occurs when positive changes in overall deprivation increase dramatically the observed deprivation in one group and produces merely positive changes in another. This could potentially mean that there is an unobserved factor affecting the relationship. In the second case, a group is systematically more deprived than other, and such a differential in deprivation is not explained by the factor.

This seems to lead to a disappointing conclusion, because in poverty research it is expected to find variations in observed deprivations across sub-populations—we should expect that a higher prevalence of food deprivation among indigenous people than among the non-indigenous population. This, however, is an incorrect interpretation. The relationship between food deprivation (latent construct) and its manifestations across groups is a rather, albeit connected, different issue. A regression model could be utilised to check the odds of being deprived between different groups. However, the difference in the odds could be due to violations of MI, which is not necessarily an undesirable outcome, *provided a predictor of the differences between groups is included, which would equate to introducing a factor in the measurement model. Otherwise, the differences would be attributed exclusively to group membership rather than to the underlying phenomenon associated with the group in question*.

Not surprisingly, the main source of MI is *selection*, which is likely to affect the relationship between the manifest variables and the factor (Aitken 1935). Thomson (1939) claimed that a measurement model will be “dependent upon the population in which they are measured” (p. 194). We are going to always find differences between groups, and it is unlikely to account for all the unobservable factors that affect the measurement model across these groups. Years later, nonetheless, Thurstone (1947) showed that it should be possible to find a *simple structure* which can be preserved after selection. This means that it is possible to test and find a model in which systematic differences between groups are exclusively due to the common factor.

As a result, theoretically, it is possible to find a simple structure or core measurement model, regardless of how large the variations in prevalence may be between groups, upon which comparisons are tenable. The key point is that the relationship between the indicators of such a model and selection are conditional exclusively on the latent construct. Both Townsend (1979) and Sen (1985) agreed that this kind of measure is more likely to facilitate comparisons across countries, and it is akin to the idea of finding a simple structure. Drawing upon this presumption, there has been some work in this regard using absolute measures, i.e. measures with severe thresholds, to compare countries (Gordon et al. 2003; Alkire and Santos 2010; Battiston et al. 2013; UNDP 2014). However, the properties of these measures have not been tested yet.^1^

As previously noted, the questionnaire is another potential source for increasing the negative effects of selection upon measurement invariance. This, in fact, is becoming an emergent topic in comparative social research in which the analysis of MI is increasingly important (Vannieuwenhuyze et al. 2010; Vannieuwenhuyze 2015). This could happen when a given question is consistently interpreted differently by sub-populations (Keith 2015). It is not that the survey systematically favours a condition of a sub-population but that the questions are interpreted consistently different between sub-groups. Although questions on capturing deprivation are mostly straightforward (dichotomous questions), things can get lost in translation across countries (for example EHLEIS 2013).

In practice, Measurement invariance involves testing whether different parameters of a measurement model are equivalent across groups. The concept of the measurement model in poverty research equates to the structure of a measure which is given by the way in which indicators are classified into different dimensions, for example consider the classic Townsend model (Townsend 1979), in which indicators are classified into material and social deprivation. Those two correspond to two dimensions of deprivation, which in turn measure a higher factor called ‘overall deprivation’. From the perspective of MI, this would mean that both dimensions are measured on the same basis across groups and that the relationship between material and social deprivation and the indicators is equivalent across groups. An example of this notion could mean asking whether not having shoes measures material deprivation invariantly in countries ‘A’ and ‘B’. In other words, changes in material deprivation should be associated with increments/decrements in the observed prevalence of not having shoes in both countries.

In the context of poverty research, is important to make a distinction between scale equating (prevalence weighting) and Measurement Invariance. From an empirical perspective, prevalence weighting (i.e. deprivation weighted by the frequency of those who have each item) gives more relative importance to those items that are more commonly possessed (Fusco et al. 2013). Because this strategy adjusted by differentials in severity of deprivation, it might be useful to smooth poverty estimates over time or across countries. Prevalence weighting can be seen as a simpler version of scale equating, which applies a constant to a set of indicators in order to adjust the item considering severity and their discriminatory capabilities (i.e. which items are more useful to identify the poor) (Kolen and Brennan 2004). However, scaling and MI are different concepts. For instance, any form of equating relies on indicators that are invariant otherwise produce spurious results (Kolen and Brennan 2004; von Davier 2010; Benson et al. 2015).

Prevalence weighting or, more generally speaking, scale equating, might be useful to reduce differences due to severity, i.e. comparing years after there have been changes in the standards of society or comparing two countries with different standards of living in the absence of an absolute measure. However, the usefulness of prevalence weighting for equating an index remains as an empirical question. The key point is that MI is a necessary condition of conducting scale equating. Multidimensional indexes utilize different indicators and there are other sources affecting the relationship between the observed items and poverty: Differences in prices, preferences and/or data collection problems (sampling, translation of questionnaires, interpretation of specific questions) are likely to affect how poverty is measured by the set of indicators.

## Measurement invariance

In order to gain a better understanding of what constitutes MI in the context of a measurement model, it is important to consider the types of parameters involved in MI testing, as well as the types of MI. Statistically, MI holds when the parameters involved in common factor analysis, such as the latent variable mean, thresholds, factor loadings and residual variances, are equivalent across populations, i.e. they do not depend on population membership (see model formulation next section) (Meredith 1993). In other words, MI implies that, conditional on the factor score, the observed scores (observed material deprivations) do not depend on population membership. That is, people with the same level of material deprivation should have, on average, the same observed deprivations. Given this condition, if the average of the observed deprivations differs between people living in different areas, it is likely that the items will measure a different construct in one area in relation to another.

In practice violations of MI are due to non-invariant thresholds (intercepts) and slopes (loadings). In the first case, non-invariant intercepts mean that for the same latent score of deprivation, people living in different states systematically have differences in observed deprivations at a given item. In the second case, different slopes between two groups, for example, suggest that for different values in the unobserved deprivation, changes in the values of the observed deprivations differ. When the latent deprivation score is low (i.e. mild deprivation), the observed deprivation in one group is higher in relation to the other.

In practice it is likely to find different degrees of MI which represent the extent through which populations are comparable (Meredith 1993; Meredith and Teresi 2006). Each type can be defined as follows:

*Strong MI* (also known as scalar) occurs when the factor loadings and the intercepts are identical across groups. In other words, the mean and the variances are non-invariant across groups. This situation was illustrated above and occurs when the slopes and the thresholds are equivalent across groups. Meredith and Teresi (2006) proved that scalar MI almost ensures MI.

*Weak or metric MI* requires the lowest equality of intercepts. Different thresholds imply systematic differences between groups, i.e. the starting point is different.^2^ It is important to mention the practical consequences of metric MI. Deprivations scores are calculated using the sum of the item. Metric MI will inflate the importance of the source of non-invariance. In other words, it is like (unintentionally) adding weight to a specific indicator, which will result in penalties given to the group that is disfavoured by including such an indicator. Therefore, the deprivation score will produce a mistaken picture of deprivation severity when comparing sub-populations.

Finally, *Configural MI* refers to the case where all parameters in the model violate MI, and comparisons between groups in the presence of this kind of MI are absolutely untenable (Meredith 1993).

## Alignment method

This article uses the alignment method to test MI (Asparouhov and Muthén 2014). Confirmatory multiple group analysis has been the main statistical approach to examining MI (van de Schoot et al. 2012; Keith 2015). There are three reasons that justify the selection of the alignment method. First, when using large samples (${\ge}500$), multiple group factor analysis will always favour the scalar model. Similarly to CFA models, absolute statistics of fit such as ${\chi ^2}$ will reject the model for large datasets (Bollen 1990). For relative statistics of fit such as RMSEA (root mean square error of approximation) and the comparative fit index (CFI), the literature suggests using different criteria for model comparison (Cheung and Rensvold 2002; Chen 2007). However, assessing MI in the context of many groups (${\ge}20$) requires specifying many partial models. A number of changes are required to consider all the modification indices that emerge from the CFA model, so it is very difficult to assess which items are the sources of MI and which groups present the most problematic measurement issues. Furthermore, in the case of many modification indices, it is very likely that eventually the model will be not be identified. Third, the mean of the factor cannot be estimated from a multi-group factor model. Therefore, it is impossible to make comparisons based on the severity of deprivation across units. This is a major drawback, as one of the goals in many research projects is to have an idea about which groups present the highest severity relative to others.

The alignment method overcomes these three limitations (Asparouhov and Muthén 2014), so it therefore suits the purposes of this article, as it permits one to assess MI for many groups (32), allows for the comparison of the factor mean across states and means the use of a large sample size is not a concern. This method is based on the configural model (all parameters are free) and avoids fitting scalar or metric models. The alignment method does not assume MI, as it can estimate the mean and the variance for each group and find the optimal MI pattern. This pattern is a solution in which the measurement of non-invariance is minimised.

The alignment method therefore uses a full unconstrained model (M0) and then finds a final model with the properties described above. These two solutions are analogous to an exploratory factor model with an un-rotated solution (M0) and a rotated EFA, where the fit of the models is not compromised (for a technical overview Asparouhov and Muthén 2014).

Consider a multiple group factor model:

1 $$y_{ipg} = v_{pg} + \lambda _{pg} \eta _{ig} + \epsilon _{ipg} $$

where p is the number of dichotomous indicators, g the number of groups, i the number of independent observations in group g and ${\eta _{ig}}$ is the latent variable. Errors are distributed normally. ${v_{pg}}$ are the intercepts and ${\lambda _{pg}}$ the factor loadings. The alignment method can estimate ${v_{pg}}$, ${\lambda _{pg}}$, ${\alpha _g}$ (mean factor) and ${\psi _g}$ (variance of the factor). In the scalar model (strict MI), the parameters are fixed across states. If scalar MI is not achieved, progressively, the constrains are relaxed (from strong MI to Weak MI). Consequently, the alignment method, in the configural model, finds ${\alpha _g}$ and ${\psi _g}$ that minimise the amount of non-invariance. The alignment method then aims to find a model in which *approximate* measurement invariance holds.

The alignment method can be estimated by using both the classical approach (maximum likelihood) or Bayesian estimation within the BSEM framework (Muthén and Muthén 2012). According to simulations, both methods show very low relative bias; however, the BSEM approach seems to outperform maximum likelihood in most conditions. This article uses the classical approach, as it offers the opportunity to incorporate sampling weights, which is a drawback in most Bayesian analyses when complex surveys are utilised.^3^

## Data

As a motivating example, data from the socio-economic household conditions module in the Mexican ‘Household Income Expenditure 2012’ (ENIGH, its acronym in Spanish) survey are utilised to calculate deprivation indicators using different thresholds and to illustrate the implications of MI for the comparison of deprivation between the country’s 32 states (INEGI-CONEVAL 2012). The ENIGH is a complex survey representing national, urban and rural areas as well as the 32 states. The sample size is 212,674 cases. In addition, data from the National Survey on the Thresholds for Multidimensional Poverty (EDUMP, its acronym in Spanish) were used as a reference for the perceptions of Mexican society on what constitute minimum standards of living (CONEVAL 2007). The EDUMP is also a complex survey with representativeness at the national, urban and rural levels (n = 10,270).

The exercise utilizes two poverty measures in order to assess changes in MI when using different thresholds for some indicators (see below). This aims to illustrate the potential problems that might arise when updating a poverty measure. The two measures are the following: The official Mexican multidimensional poverty measure (Index A) and an adjusted measure (Index B). The differences are discussed below.

### The multidimensional poverty measure

The official Mexican multidimensional poverty measure (MPM) combines – using the intersection method—direct (deprivations) and indirect (income) measures to estimate poverty (CONEVAL 2011a, 2009). For this article, only the ten direct (dichotomous) indicators are used. The dimensional structure of the MPM is a higher-order factor (poverty) measured by two domains (or dimensions): deprivation (also known as ‘social rights’ or ‘standard of living dimensions’ within the Mexican context) and welfare (income poverty) (CONEVAL 2011a). Deprivation (direct measurement) is related to a set of basic socio-economic rights set out by Mexican legislation: compulsory education, access to health and social security (minimum social protection floor), access to essential public services, food deprivation and adequate housing deprivation. The indicators employed for this measure are as follows:

- Food deprivation: people suffering two or more hunger episodes.
- Access to a minimum social protection floor: people lacking health care or social security.
- Inadequate flooring: lacking a floor made of cement, tiles or laminate.
- Inadequate roofing: lacking a roof made of cement, slab, roof with beams, tile or wood.
- Inadequate walls: lacking walls made of cement, brick, block, adobe(mud) or wood.
- Overcrowding: more than 2.5 per room.
- Access to water: lacking piped water inside the property (not necessarily the house).
- Sanitation: lacking access to sewage network.
- Lacking education: people without secondary education or who are not in education (people aged 16 or under) [see (CONEVAL 2011a for details about the normative ages for different cohorts].

It has been pointed out that Mexico’s government selected thresholds or cut-offs that capture only severe deprivation and not the level of deprivation that is unacceptable according to the current standards of Mexican society (Boltvinik 2014). There are a number of implications of this mismatch for poverty measurement. For the purposes of this article, this discrepancy is useful for assessing empirically the effect of using different thresholds on MI. As a reminder, in poverty research it is claimed that using absolute measures reduces comparability problems, as it limits between-group variability in terms of prevalence. This, however, comes at the expense of underestimating poverty and deprivation. Table 1 illustrates the difference between the cut-offs of some items selected by Mexico’s government and the standard of the consensual approach. As can be appreciated the Mexican official measure uses cut-offs that capture severer forms of deprivation in comparison with the standards of the Mexican society.

**Table 1:** Comparison of thresholds between those selected for the official multidimensional poverty measure and those suggested by Mexican society

	Official measure (cut-off not deprived)	Standard of society* (cut-off not deprived)	Official thresholds (relative severity)
Adequate housing materials			
Flooring materials	(Cement-tiled-laminate-other)	(Cement-tiled-laminate-other)	Equal
Roof materials	(Cement, slab, roof with beams, tile, wood)	(Cement or slab)	Severer deprivation
Walls	(Cement, brick, block, adobe(mud) or wood)	(Cement, brick, block)	Severer deprivation
Access to public services			
Access to water inside the property or house	Inside the property or house	Inside the house and every day	Severer deprivation
Access to independent toilet	Connection to drainage	Connection to drainage and an independent toilet connected to water	Severer deprivation
Food deprivation hunger episodes			
Food insecurity	Moderate food insecurity (less 3 hunger episodes)	No food insecurity	Severer deprivation

Two indexes were produced using these different thresholds: A (official measure) and B (adjusted). For the other indicators, the threshold was the same. It is important to mention that the adjusted measure has been empirically validated following Gordon (2010), and the grouping of the indicators for index B coincided with the grouping of socially perceived necessities.

## Descriptive analysis

Table 2 shows the percentage of the total population deprived in a given item. Deprivation increases for Index B. This is due to the fact that using a less severe threshold (i.e. a cut-off associated with mild deprivation) increases the prevalence of deprivation. As can be appreciated. 62 % of the Mexican population lacks minimum social protection floor, i.e. social security and health care. Food deprivation almost doubles when using the adjusted threshold. Deprivation associated with dwelling (lacking adequate materials) increases substantially once more robust materials are considered. Water and sanitation also present an important jump. This is due to the fact that a half of the Mexican population lack access within the house every day and access to a toiled inside the house with running water.

**Table 2:** Percentage of the population deprived in relation to the given item. Mexico, 2012

	Index A (official thresholds)	Index B (adjusted)
Education	19	19
Food deprivation	23	44
Minimum social protection floor	62	62
Flooring material	4	4
Roofing material	2	25
Walling material	2	14
Overcrowding	10	10
Access to water	9	47
Sanitation	9	40
Fuel	13	13

*Source* Estimations based on INEGI-CONEVAL (2012)

**Fig. 1:**
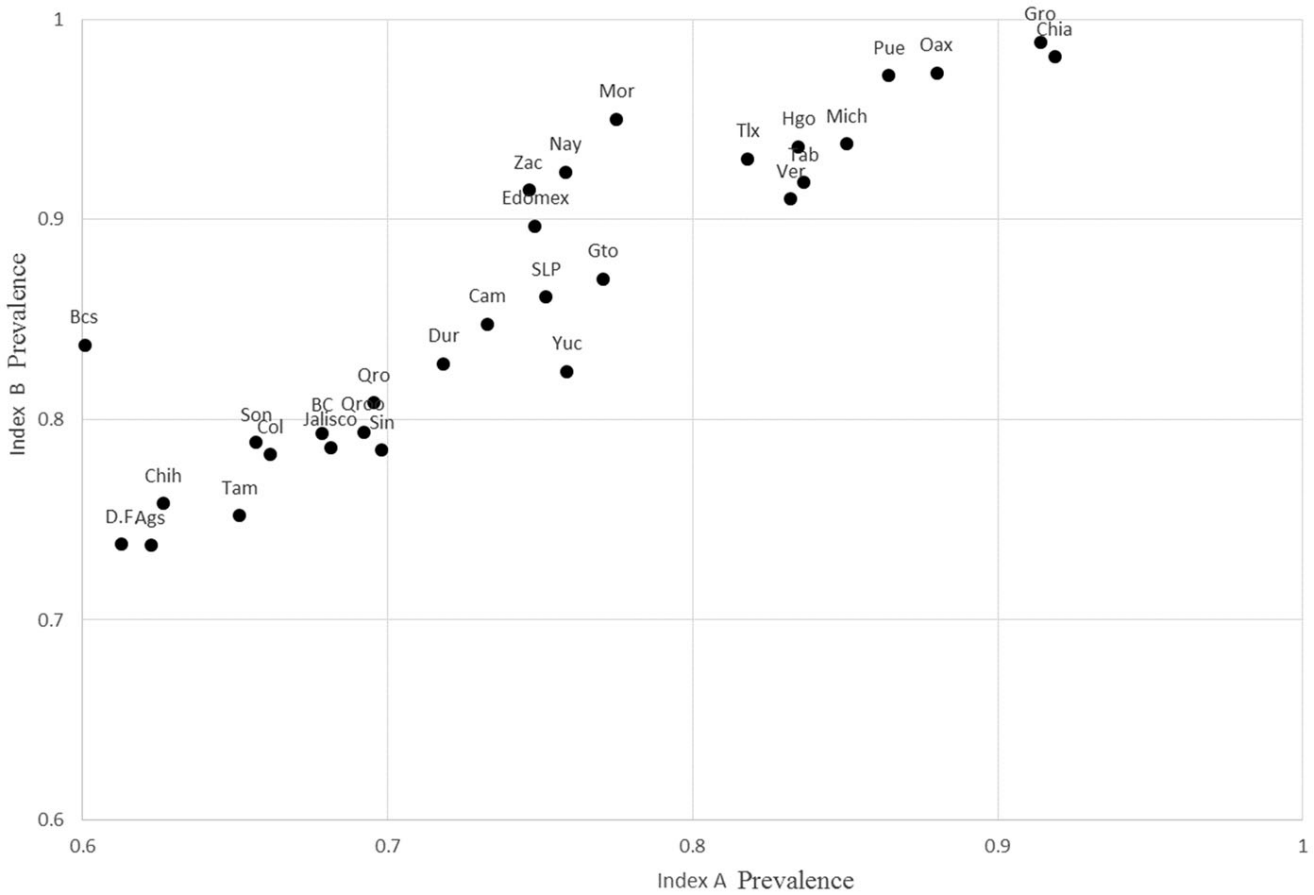
Prevalence rate. Index A versus Index B. Mexico States, 2012. *Source* Estimates produced using INEGI-CONEVAL ENIGH (2012)

Figure 1 compares variations in the prevalence of deprivation by using index ’A’ and index ’B’. Prevalence was computed using one or more deprivations as cut-off, this following the official measure. The points tend to be skewed toward the top because, understandably, Index B leads to higher prevalence rates. Using these indexes with more severe thresholds, Chiapas has the highest deprivation incidence, followed very closely by Guerrero. Baja California Sur (Bcs), Mexico City (DF) and Aguascalientes (AGS) have the lowest percentage of deprived population. When using the index with less severe thresholds (Index B,) Guerrero (Gro) has the highest percentage of people with one or more material deprivations, and Chiapas (Chip) has the second highest. Mexico City (DF) and Aguascalientes (Ags) have the lowest prevalence rates.

**Fig. 2:**
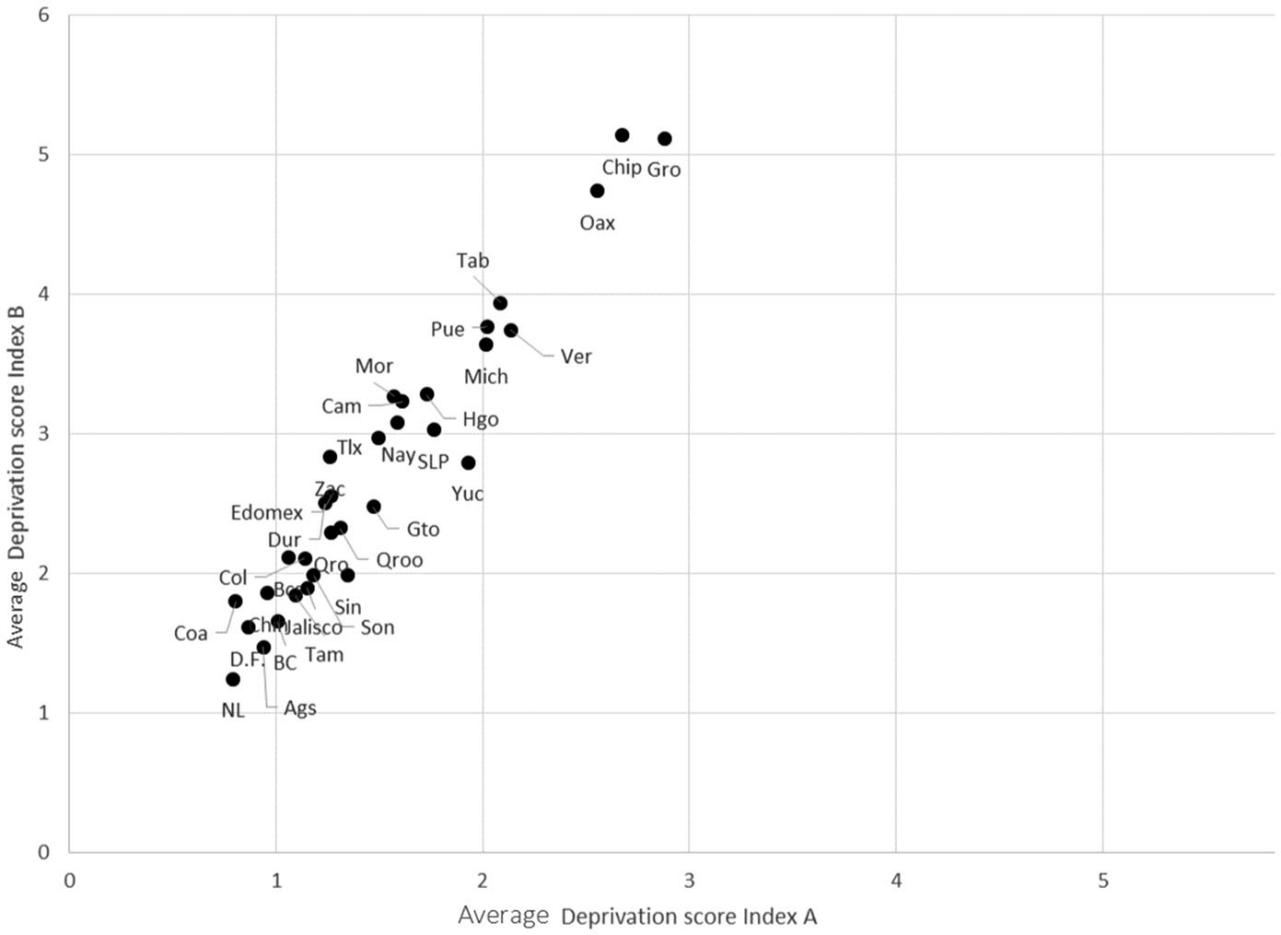
Mean severity of deprivation (average deprivation score). Index A versus Index B. Mexico. States, 2012. *Source* Estimates produced using INEGI-CONEVAL ENIGH (2012)

Figure 2 contrasts the average deprivation score, using index ‘A’ and index ‘B’. As expected, the average deprivation score rises across all states using index ‘B’, due to the use of more severe thresholds. There are changes in the ordering of states with lower deprivation scores, meaning that different thresholds most certainly affect ranking.

## Results

The analysis of MI was conducted using the alignment method. Maximum likelihood was utilized in order to incorporate the design of the survey. According to the analysis, Partial strong MI holds for the official index (Table 3, Index A). All of the slopes are equal across states and, leaving aside walling materials and fuel, all the items show few states (less than five) with non-invariant thresholds. Overall, this suggest that the Index A leads to robust comparisons across states. The fact that two indicators show problems could imply the need to drop them. However, before taking such a drastic decision is important to revise whether this leads to significant differences (Tables 4, 6). There is no strong evidence to conclude that these indicators lead to acute classification problems. Therefore, if the measure is utilized to inform specific policies, the advise would be to keep them as long as the model-based estimates are supplied and interpreted (see below).

Partial, very weak MI (only loadings are MI) is present in the case of the second index (Table 3, Index B). In this case, under rigorous criteria, it could be concluded that this index is non-invariant, given that some items have unequal intercepts and slopes for several states. In particular, the loadings of the indicator for access to water are not equal for eleven states. This is not a good indicator for making comparisons between states. It is likely that this is due to the use of a very high threshold, whereby states with discrepancies in the value of the intercept tend to be those with higher deprivation rates. Although this argument seems to be valid for the data used in this article, further Monte Carlo experiments need to be undertaken to assess further how aggregation—and in particular dimensionality—affects the behaviour of the alignment method.

**Table 3:** Number of states with strong comparability problems. Number of non-invariant intercepts and factor loadings

	Index A	Index B
Intercepts		
Food deprivation	5	6
Minimum social protection floor	4	6
Basic education deprivation	0	10
Overcrowding	2	4
Adequate walling materials	12	13
Adequate flooring	1	3
Adequate roofing	0	16
Adequate fuel	9	15
Access to water	4	12
Sanitation	0	2
Loadings		
Food deprivation	0	0
Minimum social protection floor	0	2
Basic education deprivation	0	0
Overcrowding	0	0
Adequate walling materials	0	1
Adequate flooring	0	0
Adequate roofing	0	2
Adequate fuel	0	4
Access to water	0	19
Sanitation	0	1

*Source* Estimates using INEGI-CONEVAL (2012). Alignment method

Estimates adjusted by sampling weights

In the case of the intercepts for Index B, the access to water indicator presents substantive differences for more than half of the states. Many of the indicators that were adjusted using the standards of Mexican society show important disparities in the value of the intercepts across states: walling materials, roofing materials and roofing materials. This is not the case for sanitation. This raises the question on the use of a national standard in a highly unequal country, without considering whether the standard varies across different geographical units. The extent of the problem has to be assessed in terms of what type or level of MI is reasonable in a given study. Regarding this issue, the analysis suggests that in order to make more solid comparisons between states, it would be highly advisable to at least attain weak measurement invariance (i.e. slope equality) by adjusting the threshold of the access to water indicator. This of course should be normatively and theoretically informed, as the adjustment would affect any estimation of the extent of poverty.

If no changes are undertaken, by using the alignment method it is possible to estimate factor means in the presence of approximate invariance. Tables 4 and 5 compare, using a significance test, the mean value of deprivation per state for both indexes, i.e. index A and B, respectively. According to Table 4, the state with severest level of material deprivation is Chiapas (7). However, no statistical differences were found between this state and Guerrero (12). As can be appreciated, there are no major differences in the mean deprivation score between the states with the lowest values, which suggests that the mean level of deprivation tends to be very similar between these states.

**Table 4:** Model-based ranking. Index A. States ranked from high to low severity

	7	12	20	30	27	16	21	13	31	11	24	4	17	9	32	18
7	X		$>$	$>$	$>$	$>$	$>$	$>$	$>$	$>$	$>$	$>$	$>$	$>$	$>$	$>$
12		X		$>$	$>$	$>$	$>$	$>$	$>$	$>$	$>$	$>$	$>$	$>$	$>$	$>$
20	$<$		X					$>$	$>$	$>$	$>$	$>$	$>$	$>$	$>$	$>$
30	$<$	$<$		X						$>$	$>$	$>$	$>$	$>$	$>$	
27	$<$	$<$			X					$>$	$>$	$>$	$>$	$>$	$>$	
16	$<$	$<$				X					$>$	$>$	$>$	$>$	$>$	
21	$<$	$<$					X					$>$	$>$		$>$	
13	$<$	$<$	$<$					X								
31	$<$	$<$	$<$						X							
11	$<$	$<$	$<$	$<$	$<$					X						
24	$<$	$<$	$<$	$<$	$<$	$<$					X					
4	$<$	$<$	$<$	$<$	$<$	$<$	$<$					X				
17	$<$	$<$	$<$	$<$	$<$	$<$	$<$						X			
9	$<$	$<$	$<$	$<$	$<$	$<$								X		
32	$<$	$<$	$<$	$<$	$<$	$<$	$<$								X	
18	$<$	$<$	$<$													X
29	$<$	$<$	$<$													
23	$<$	$<$	$<$	$<$	$<$	$<$	$<$	$<$	$<$	$<$		$<$				
22	$<$	$<$	$<$	$<$	$<$	$<$	$<$	$<$	$<$	$<$	$<$	$<$				
6	$<$	$<$	$<$	$<$	$<$	$<$	$<$	$<$	$<$	$<$	$<$	$<$				
14	$<$	$<$	$<$	$<$	$<$	$<$	$<$	$<$	$<$	$<$	$<$	$<$				
10	$<$	$<$	$<$	$<$	$<$	$<$	$<$	$<$	$<$	$<$	$<$	$<$				
25	$<$	$<$	$<$	$<$	$<$	$<$	$<$	$<$	$<$	$<$	$<$					
15	$<$	$<$	$<$	$<$	$<$	$<$	$<$	$<$	$<$	$<$	$<$					
28	$<$	$<$	$<$	$<$	$<$	$<$	$<$	$<$	$<$	$<$	$<$	$<$	$<$			
26	$<$	$<$	$<$	$<$	$<$	$<$	$<$	$<$	$<$	$<$	$<$	$<$	$<$			
3	$<$	$<$	$<$	$<$	$<$	$<$	$<$	$<$	$<$	$<$	$<$	$<$	$<$			
2	$<$	$<$	$<$	$<$	$<$	$<$	$<$	$<$	$<$	$<$	$<$	$<$	$<$	$<$	$<$	
1	$<$	$<$	$<$	$<$	$<$	$<$	$<$	$<$	$<$	$<$	$<$	$<$	$<$			
5	$<$	$<$	$<$	$<$	$<$	$<$	$<$	$<$	$<$	$<$	$<$	$<$	$<$	$<$	$<$	
8	$<$	$<$	$<$	$<$	$<$	$<$	$<$	$<$	$<$	$<$	$<$	$<$	$<$			
19	$<$	$<$	$<$	$<$	$<$	$<$	$<$	$<$	$<$	$<$	$<$	$<$	$<$	$<$	$<$	

	29	23	22	6	14	10	25	15	28	26	3	2	1	5	8	19
7	$>$	$>$	$>$	$>$	$>$	$>$	$>$	$>$	$>$	$>$	$>$	$>$	$>$	$>$	$>$	$>$
12	$>$	$>$	$>$	$>$	$>$	$>$	$>$	$>$	$>$	$>$	$>$	$>$	$>$	$>$	$>$	$>$
20	$>$	$>$	$>$	$>$	$>$	$>$	$>$	$>$	$>$	$>$	$>$	$>$	$>$	$>$	$>$	$>$
30		$>$	$>$	$>$	$>$	$>$	$>$	$>$	$>$	$>$	$>$	$>$	$>$	$>$	$>$	$>$
27		$>$	$>$	$>$	$>$	$>$	$>$	$>$	$>$	$>$	$>$	$>$	$>$	$>$	$>$	$>$
16		$>$	$>$	$>$	$>$	$>$	$>$	$>$	$>$	$>$	$>$	$>$	$>$	$>$	$>$	$>$
21		$>$	$>$	$>$	$>$	$>$	$>$	$>$	$>$	$>$	$>$	$>$	$>$	$>$	$>$	$>$
13		$>$	$>$	$>$	$>$	$>$	$>$	$>$	$>$	$>$	$>$	$>$	$>$	$>$	$>$	$>$
31		$>$	$>$	$>$	$>$	$>$	$>$	$>$	$>$	$>$	$>$	$>$	$>$	$>$	$>$	$>$
11		$>$	$>$	$>$	$>$	$>$	$>$	$>$	$>$	$>$	$>$	$>$	$>$	$>$	$>$	$>$
24			$>$	$>$	$>$	$>$	$>$	$>$	$>$	$>$	$>$	$>$	$>$	$>$	$>$	$>$
4		$>$	$>$	$>$	$>$	$>$			$>$	$>$	$>$	$>$	$>$	$>$	$>$	$>$
17									$>$	$>$	$>$	$>$	$>$	$>$	$>$	$>$
9												$>$		$>$		$>$
32												$>$		$>$		$>$
18																
29	X															
23		X										$>$		$>$		$>$
22			X									$>$		$>$		$>$
6				X										$>$		$>$
14					X									$>$		$>$
10						X								$>$		$>$
25							X							$>$		$>$
15								X						$>$		$>$
28									X					$>$		$>$
26										X						
3											X					
2		$<$	$<$									X				
1													X			
5		$<$	$<$	$<$	$<$	$<$	$<$	$<$	$<$					X		
8															X	
19		$<$	$<$	$<$	$<$	$<$	$<$	$<$	$<$							X

$<,$ Significantly lower deprivation; $>,$ significantly higher

Table 5 displays the ranking obtained for index B after applying the alignment method and in the light of significance tests on the mean factor value (i.e. severity). As can be appreciated, there are more significant differences between states in comparison with Table 4. There are no differences between states 7 and 12, which are the two with the highest levels of severity. If model-based estimates are going to be utilised to produce rankings, these significant differences need to be taken into consideration.

**Table 5:** Model-based ranking. Index B. States ranked from high to low severity

	7	12	20	27	21	30	16	13	17	4	24	18	32	29	11	31
7	X		$>$	$>$	$>$	$>$	$>$	$>$	$>$	$>$	$>$	$>$	$>$	$>$	$>$	$>$
12		X		$>$	$>$	$>$	$>$	$>$	$>$	$>$	$>$	$>$	$>$	$>$	$>$	$>$
20	$<$		X		$>$	$>$	$>$	$>$	$>$	$>$	$>$	$>$	$>$	$>$	$>$	$>$
27	$<$	$<$		X				$>$	$>$	$>$	$>$	$>$	$>$	$>$	$>$	$>$
21	$<$	$<$	$<$		X				$>$	$>$	$>$	$>$	$>$	$>$	$>$	$>$
30	$<$	$<$	$<$			X			$>$	$>$	$>$	$>$	$>$	$>$	$>$	$>$
16	$<$	$<$	$<$				X			$>$			$>$	$>$	$>$	$>$
13	$<$	$<$	$<$	$<$				X						$>$		$>$
17	$<$	$<$	$<$	$<$	$<$	$<$			X							
4	$<$	$<$	$<$	$<$	$<$	$<$	$<$			X						
24	$<$	$<$	$<$	$<$	$<$	$<$					X					
18	$<$	$<$	$<$	$<$	$<$	$<$						X				
32	$<$	$<$	$<$	$<$	$<$	$<$	$<$						X			
29	$<$	$<$	$<$	$<$	$<$	$<$	$<$	$<$						X		
11	$<$	$<$	$<$	$<$	$<$	$<$	$<$								X	
31	$<$	$<$	$<$	$<$	$<$	$<$	$<$	$<$								X
9	$<$	$<$	$<$	$<$	$<$	$<$	$<$									
10	$<$	$<$	$<$	$<$	$<$	$<$	$<$	$<$	$<$							
15	$<$	$<$	$<$	$<$	$<$	$<$	$<$	$<$	$<$	$<$						
22	$<$	$<$	$<$	$<$	$<$	$<$	$<$	$<$	$<$	$<$				$<$		
6	$<$	$<$	$<$	$<$	$<$	$<$	$<$	$<$	$<$	$<$	$<$	$<$	$<$	$<$	$<$	
14	$<$	$<$	$<$	$<$	$<$	$<$	$<$	$<$	$<$	$<$	$<$	$<$	$<$	$<$	$<$	$<$
23	$<$	$<$	$<$	$<$	$<$	$<$	$<$	$<$	$<$	$<$	$<$	$<$	$<$	$<$	$<$	$<$
25	$<$	$<$	$<$	$<$	$<$	$<$	$<$	$<$	$<$	$<$	$<$	$<$	$<$	$<$	$<$	$<$
5	$<$	$<$	$<$	$<$	$<$	$<$	$<$	$<$	$<$	$<$	$<$	$<$	$<$	$<$	$<$	$<$
3	$<$	$<$	$<$	$<$	$<$	$<$	$<$	$<$	$<$	$<$	$<$	$<$	$<$	$<$	$<$	$<$
28	$<$	$<$	$<$	$<$	$<$	$<$	$<$	$<$	$<$	$<$	$<$	$<$	$<$	$<$	$<$	$<$
1	$<$	$<$	$<$	$<$	$<$	$<$	$<$	$<$	$<$	$<$	$<$	$<$	$<$	$<$	$<$	$<$
26	$<$	$<$	$<$	$<$	$<$	$<$	$<$	$<$	$<$	$<$	$<$	$<$	$<$	$<$	$<$	$<$
2	$<$	$<$	$<$	$<$	$<$	$<$	$<$	$<$	$<$	$<$	$<$	$<$	$<$	$<$	$<$	$<$
8	$<$	$<$	$<$	$<$	$<$	$<$	$<$	$<$	$<$	$<$	$<$	$<$	$<$	$<$	$<$	$<$
19	$<$	$<$	$<$	$<$	$<$	$<$	$<$	$<$	$<$	$<$	$<$	$<$	$<$	$<$	$<$	$<$

	9	10	15	22	6	14	23	25	5	3	28	1	26	2	8	19
7	$>$	$>$	$>$	$>$	$>$	$>$	$>$	$>$	$>$	$>$	$>$	$>$	$>$	$>$	$>$	$>$
12	$>$	$>$	$>$	$>$	$>$	$>$	$>$	$>$	$>$	$>$	$>$	$>$	$>$	$>$	$>$	$>$
20	$>$	$>$	$>$	$>$	$>$	$>$	$>$	$>$	$>$	$>$	$>$	$>$	$>$	$>$	$>$	$>$
27	$>$	$>$	$>$	$>$	$>$	$>$	$>$	$>$	$>$	$>$	$>$	$>$	$>$	$>$	$>$	$>$
21	$>$	$>$	$>$	$>$	$>$	$>$	$>$	$>$	$>$	$>$	$>$	$>$	$>$	$>$	$>$	$>$
30	$>$	$>$	$>$	$>$	$>$	$>$	$>$	$>$	$>$	$>$	$>$	$>$	$>$	$>$	$>$	$>$
16	$>$	$>$	$>$	$>$	$>$	$>$	$>$	$>$	$>$	$>$	$>$	$>$	$>$	$>$	$>$	$>$
13		$>$	$>$	$>$	$>$	$>$	$>$	$>$	$>$	$>$	$>$	$>$	$>$	$>$	$>$	$>$
17		$>$	$>$	$>$	$>$	$>$	$>$	$>$	$>$	$>$	$>$	$>$	$>$	$>$	$>$	$>$
4			$>$	$>$	$>$	$>$	$>$	$>$	$>$	$>$	$>$	$>$	$>$	$>$	$>$	$>$
24					$>$	$>$	$>$	$>$	$>$	$>$	$>$	$>$	$>$	$>$	$>$	$>$
18					$>$	$>$	$>$	$>$	$>$	$>$	$>$	$>$	$>$	$>$	$>$	$>$
32					$>$	$>$	$>$	$>$	$>$	$>$	$>$	$>$	$>$	$>$	$>$	$>$
29				$>$	$>$	$>$	$>$	$>$	$>$	$>$	$>$	$>$	$>$	$>$	$>$	$>$
11					$>$	$>$	$>$	$>$	$>$	$>$	$>$	$>$	$>$	$>$	$>$	$>$
31						$>$	$>$	$>$	$>$	$>$	$>$	$>$	$>$	$>$	$>$	$>$
9	X						$>$	$>$	$>$	$>$	$>$	$>$	$>$	$>$	$>$	$>$
10		X							$>$	$>$	$>$	$>$	$>$	$>$	$>$	$>$
15			X						$>$	$>$	$>$	$>$	$>$	$>$	$>$	$>$
22				X					$>$	$>$	$>$	$>$	$>$	$>$	$>$	$>$
6					X				$>$	$>$	$>$	$>$	$>$	$>$	$>$	$>$
14						X						$>$		$>$	$>$	$>$
23	$<$						X					$>$		$>$	$>$	$>$
25	$<$							X						$>$	$>$	$>$
5	$<$	$<$	$<$	$<$	$<$				X						$>$	$>$
3	$<$	$<$	$<$	$<$	$<$					X					$>$	$>$
28	$<$	$<$	$<$	$<$	$<$						X					$>$
1	$<$	$<$	$<$	$<$	$<$	$<$	$<$					X				
26	$<$	$<$	$<$	$<$	$<$								X			
2	$<$	$<$	$<$	$<$	$<$	$<$	$<$	$<$						X		
8	$<$	$<$	$<$	$<$	$<$	$<$	$<$	$<$	$<$	$<$					X	
19	$<$	$<$	$<$	$<$	$<$	$<$	$<$	$<$	$<$	$<$	$<$					X

$<,$ Significantly lower deprivation ; $>,$ significantly higher

Table 6 ranks the 32 Mexican states according to both the mean deprivation score and the model-based estimates using Index A. Although there are some states with dramatic changes in their position, when comparing both estimates of severity, there are no significant differences according to Table 4. For example, for Tlx, its severity is lower than states 12, 7 and 20 and equal to the rest. Edomex could be ranked between 10 and 26, while Bcs could be ranked as state 13 or above. The only exception is the Federal District (DF). According to the alignment method there are three states with lower levels of severity. This differs slightly f the position assigned to this state using the deprivation score (30th) (Table 6). Overall, this supports the hypothesis that when scalar MI holds, it is possible to make valid and reliable comparisons across states.

**Table 6:** Index A. States ranked according to their mean deprivation score and the model-based estimates. Mexico, 2012

State (code)	Deprivation Score	Model-based
Gro,12	1	2
Chip,7	2	1
Oax,20	3	3
Pue,21	4	7
Ver,30	5	4
Yuc,31	6	9
Mich,16	7	6
Tab,27	8	5
Hgo,13	9	8
Mor,17	10	13
SLP,24	11	11
Tlx,29	12	17
Cam,4	13	12
Nay,18	14	16
Edomex,15	15	24
Zac,32	16	15
Gto,11	17	10
Qro,22	18	19
Qroo,23	19	18
Sin,25	20	23
Bcs,3	21	27
Dur,10	22	22
Jalisco,14	23	21
Col,6	24	20
Tam,28	25	25
Son,26	26	26
Coa,5	27	30
BC,2	28	28
Ags,1	29	29
DF,9	30	14
Chih,8	31	31
NL,19	32	32

Table 7 shows the same comparison as Table 6. It ranks states according to the two measures of severity of deprivation. Yuc (31), one of the states with unequal loadings and thresholds, should be classified 8th or above and not 6 as suggested by the mean deprivation score. Gto (11) is ranked 18th according to the deprivation score, but it could be classified between places 8th and 17th, using the alignment method. Bcs 3 is just classified in 22nd place, which is the minimum value suggested by the model-based estimates. It seems that weak MI increases discrepancies between model-based estimates and the deprivation score. However, by using a significance test it is possible to assess to what extent such discrepancies result in a different ordering. In this case it seems that weak MI does not substantially affect the ranking of the states, which seems to be due to the fact that only the access to water indicator shows non-invariant thresholds and loadings.

**Table 7:** Index B. States ranked according to their mean deprivation score and model-based estimates. Mexico, 2012

State (code)	Deprivation score	Model-based
Chip,7	1	1
Gro,12	2	2
Oax,20	3	3
Tab,27	4	5
Pue,21	5	4
Ver,30	6	7
Mich,16	7	6
Cam,4	8	14
Hgo,13	9	8
Yuc,31	10	10
SLP,24	11	16
Mor,17	12	12
Nay,18	13	13
Zac,32	14	15
Tlx,29	15	11
Edomex,15	16	17
Dur,10	17	20
Gto,11	18	9
Qroo,23	19	18
Qro,22	20	19
Sin,25	21	23
Bcs,3	22	29
Col,6	23	25
Coa,5	24	31
Tam,28	25	24
Jalisco,14	26	21
Son,26	27	26
Chih,8	28	30
BC,2	29	28
D.F.,9	30	22
Ags,1	31	27
NL,19	32	32

## Conclusions and discussion

The article discusses and illustrates the importance of testing for measurement invariance (MI) in poverty research, and it uses the Mexican multidimensional poverty measure (MPM, Index A) and a modified index based on less severe thresholds (Index B) as motivating examples. The results suggest that partial strong MI holds for the MPM (Index A), which means that valid comparisons can be made across the 32 Mexican states. Partial MI indicates that there are some indicators with invariant loadings and they need to be subject to scrutiny. The indicators of walling materials and fuel deprivation show a considerable number of states with non-invariant loadings. Partial strong MI should be enough to make valid comparisons, however, if one indicator is invariant across many units it might have a higher impact upon poverty estimates. Researchers are encouraged to assess these effects by removing the indicator from the analysis and comparing the model-based estimates of deprivation with the ranking based on a deprivation score. This will provide an idea of the extent of the problem and inform whether that indicators should be dropped from the analysis.

The index based on less severe thresholds (Index B) shows partial weak measurement invariance. The main source of non-invariance is access to water. Interestingly, adjusting the cut-offs seems to result in an increase in violations of MI. Walling materials and roofing show strong signs of non-invariant thresholds, thus highlighting that in certain states there is a systematic difference in such indicators that is not accounted for by the factor. Failing to achieve strong MI is likely to have important consequences for the computation and interpretation of deprivation scores based on the sum of deprivation, and it could inflate the importance of certain indicators, which in turn will penalise the states in which such indicators are non-invariant. For Index B, the effect of weak MI on the ordering of the states according to their severity does not seem to be very important.

A second point relates to a wider discussion about what constitutes an acceptable level of invariance in poverty research. For Meredith and Teresi (2006), this relates to the purpose of a scale. It is clear that the objective should be to achieve strong MI, but researchers might be tempted to accept softer versions (i.e. one problematic item, a small set of units showing problems). If a decision is made in this direction, the alignment method provides enough information to assess to what extent the model-based estimates of severity differ from the ordering obtained without adjusting for MI. This is, nevertheless, a non-trivial comparison, as it is uncertain which is the most reliable and valid way to measure severity (Alkire and Santos 2013; Delamonica and Minujin 2007; Atkinson 2003). Strong or partial strong MI therefore, should be a goal in comparative poverty analysis. If data using different data collection modes are utilized to produce deprivation indexes, the advise is to at least guarantee weak MI (Metric MI).

One practical question may be asked at this point: how important is it to use model-based estimates of severity when contrasting subgroups? When model-based estimates and the deprivation score lead to the same results, and no violations of MI are found, this is an indication that the metric of the deprivation score is likely to be correct. Since this is a result of the plain sum of the items, it will offer support to the underlying assumptions of such a procedure, i.e. items are additive and should have the same weight. This is not a minor issue, as one of the main concerns in poverty studies is about weighting and combining indicators to produce a score (Decancq and Lugo 2013; Alkire and Foster 2011b; Boltvinik 1998).

This article focused the analysis on comparisons between geographical units. However, the literature about MI underlines the importance of fulfilling MI when comparing groups. In poverty research is fundamental to consider the role that socially perceived needs and choice might have upon comparability. Socially perceived necessities produce an aggregate estimate of the standards of society. As has been acknowledged in the literature Mack et al. (2013), it is important to assess differences in perceived needs across population groups. Therefore, comparisons are conducted on a more solid basis. It would be a matter of future research to assess how similar perceived needs translate into meeting MI. However, due to the fact, that there are many other sources of MI (measurement error), it is critical to consider these other factors.

Regarding group comparisons, often in poverty research, follow-up analyses based on regression models utilise a poverty index as the response variable to investigate further differences across groups ( for example Ayllón 2014; Marx and Nolan 2012; Berthoud and Bryan 2011; Vandecasteele 2011; Whelan et al. 2010; Halleröd and Larsson 2008). However, there are question that have been seldom discussed such as: whether and under which circumstances a comparable measure is desirable, what could be the implications of lacking a comparable measure, and how a comparable measure can be tested and produced. In presence of weak MI is advisable to consider the potential implications of violations of MI upon the findings. It is vital to distinguish between the effects of non-invariance and group membership.

The paper aimed at underlying the importance of MI in poverty research. In this regard, it is vital to distinguish between scaling a measure (prevalence weighting and scale equating methods), and analysing MI. While scaling is useful to account for by severity and produce smooth estimates, this procedure is only reliable in as much as the indicators fulfil MI. There an important gap in poverty research in this regard as no much is about how well prevalence weighting enables to adjust poverty time-series, for example. Exercises are required to assess how prevalence weighting behaves under violations of MI and when MI is guaranteed.

Assumptions regarding MI should be explicitly stated and tested when comparisons across groups or years are the objective of a study, as they are likely to affect conclusions about the severity of poverty and deprivation. Alkire and Roche (2011)’s *disaggregation* term must be distinguished from the statistical assumption of invariance. While the former can be useful for pointing out a practical feature of a measure, the second has wider implications for the scope and use of a given index. Disaggregation might be a possibility, but invariance is essential for comparisons to be valid.

## Footnotes

1 Mack et al. (2013) found that there is a general consensus on the minimum standard of living in Britain across several groups. Does this guarantee that MI holds for such groups in the UK? Although these findings point in the right direction, it not sufficient to assume measurement equivalence. This is evidence of an agreement on the standard of living, not empirical evidence about the relationship between deprivation and its manifestations. From a statistical point of view, selection affects the covariance structure (i.e. the relationship between overall deprivation and the manifest variables) and even the dimensional structure of the measure (Aitken 1935; Thurstone 1947); therefore, without testing, it is impossible to conclude whether the *simple structure* holds.

2 This is illustrated by Brown (2006), using the agoraphobia example. Unaccounted factors might affect groups differently, and so the intercepts will be different—not due to group membership but due to such unobserved factors.

3 There are two estimation options: fixed and free. The former fixes the mean of the first group to zero (reference group). In contrast, ${\alpha _1}$ is a parameter that needs to be estimated using the free approach. For all models in this paper, the fixed approach (reference group) was utilised, because experimental evidence suggests that the free alignment approach leads to less reliable results in comparison to the fixed method (Asparouhov and Muthén 2014)
